# The InBIO Barcoding Initiative Database: contribution to the knowledge of DNA barcodes of the vascular plants of north-eastern Portugal

**DOI:** 10.3897/BDJ.13.e142020

**Published:** 2025-01-24

**Authors:** João Queirós, Rodrigo Silva, Catarina J. Pinho, Hélia M. Vale-Gonçalves, Ricardo Pita, Paulo C. Alves, Pedro Beja, Joana Paupério, Miguel Porto

**Affiliations:** 1 CIBIO, Centro de Investigação em Biodiversidade e Recursos Genéticos, InBIO Laboratório Associado, Campus de Vairão, Universidade do Porto, 4485-661 Vairão, Vila do Conde, Portugal CIBIO, Centro de Investigação em Biodiversidade e Recursos Genéticos, InBIO Laboratório Associado Campus de Vairão, Universidade do Porto, 4485-661 Vairão, Vila do Conde Portugal; 2 BIOPOLIS, Program in Genomics, Biodiversity and Land Planning, CIBIO, Campus de Vairão, 4485-661 Vairão, Vila do Conde, Portugal BIOPOLIS, Program in Genomics, Biodiversity and Land Planning, CIBIO Campus de Vairão, 4485-661 Vairão, Vila do Conde Portugal; 3 Departamento de Biologia, Faculdade de Ciências da Universidade do Porto, Rua do Campo Alegre s/n, 4169-007 Porto, Portugal Departamento de Biologia, Faculdade de Ciências da Universidade do Porto Rua do Campo Alegre s/n, 4169-007 Porto Portugal; 4 Mértola Biological Station, 7750-329, Mértola, Portugal Mértola Biological Station 7750-329, Mértola Portugal; 5 LEFT, Laboratório de Ecologia Fluvial e Terrestre, UTAD, Vila Real, Portugal LEFT, Laboratório de Ecologia Fluvial e Terrestre UTAD, Vila Real Portugal; 6 CITAB-Inov4Agro, Centro de Investigação e Tecnologias Agroambientais e Biológicas, Universidade de Trás-os-Montes e Alto Douro (UTAD), 5000-801 Vila Real, Portugal CITAB-Inov4Agro, Centro de Investigação e Tecnologias Agroambientais e Biológicas Universidade de Trás-os-Montes e Alto Douro (UTAD), 5000-801 Vila Real Portugal; 7 Instituto Mediterrâneo para a Agricultura, Ambiente e Desenvolvimento (MED), Universidade de Évora, Pólo da Mitra, 7002-554 Évora, Portugal Instituto Mediterrâneo para a Agricultura, Ambiente e Desenvolvimento (MED) Universidade de Évora, Pólo da Mitra, 7002-554 Évora Portugal; 8 Mértola Biological Station, 7750-329 Mértola, Portugal Mértola Biological Station 7750-329 Mértola Portugal; 9 BIOPOLIS Program in Genomics, Biodiversity and Land Planning, CIBIO, Campus de Vairão, 4485-661 Vairão, Vila do Conde, Portugal BIOPOLIS Program in Genomics, Biodiversity and Land Planning, CIBIO Campus de Vairão, 4485-661 Vairão, Vila do Conde Portugal; 10 Current address: European Molecular Biology Laboratory, European Bioinformatics Institute, Wellcome Genome Campus, Hinxton, CB10 1SD, United Kingdom Current address: European Molecular Biology Laboratory, European Bioinformatics Institute Wellcome Genome Campus, Hinxton, CB10 1SD United Kingdom; 11 CIBIO, Centro de Investigação em Biodiversidade e Recursos Genéticos, InBIO Laboratório Associado, Instituto Superior de Agronomia, Universidade de Lisboa, 1349-017 Lisboa,, Portugal CIBIO, Centro de Investigação em Biodiversidade e Recursos Genéticos, InBIO Laboratório Associado Instituto Superior de Agronomia, Universidade de Lisboa, 1349-017 Lisboa, Portugal

**Keywords:** reference database, DNA metabarcoding, ITS2, TrnL-ef, Trnl-gh, Trás-os-Montes Region, HTS technology

## Abstract

**Background:**

Metabarcoding is invaluable for understanding trophic interactions, enabling high-resolution and rapid dietary assessments. However, it requires a robust DNA barcode reference library for accurate taxa identification. This dataset has been generated in the framework of the InBIO Barcoding Initiative (IBI) and Agrivole project. The integration of these two projects was crucial, as Agrivole aimed to investigate the trophic niche of small mammals in Trás-os-Montes Region through DNA metabarcoding, which required a reliable plant DNA barcode library for this same region. Given the large number of species not yet represented in international databases, a survey of local plants was essential to fill this gap. Thus, this study created an accurate DNA reference database for the plants of the Trás-os-Montes Region of Portugal.

**New information:**

The current DNA reference database contains 632 vascular plant samples, all morphologically identified and belonging to 435 species. This represents 14% and 38.7% of the total known plant species for Portugal and the study area, respectively.

Of the 1781 barcode sequences provided in this dataset, 1099 contain new information (61.7%) at different levels: 254 (13.6%, ITS2: 41, trnL-ef: 126, trnL-gh: 87) are completely new to GenBank and/or BOLD databases at the time of publication, 438 (24.6%, ITS2: 59, trnL-ef: 173, trnL-gh: 206) are new records for a given species and 407 (22.9%, ITS2: 187, trnL-ef: 206, trnL-gh: 14) provide additional information (e.g. different bp length, intraspecific genetic variability); the remaining 682 sequences (38.3%) are equal (100% identity) to sequences already publicly available for the identified species. Overall, this dataset represents a significant contribution to the genetic knowledge of vascular plants represented in public libraries. This is one of the public releases of the IBI database, which provides genetic and distributional data for several taxa.

All vouchers are deposited in the Herbarium of the Museum of Natural History and Science of the University of Porto (MHNC-UP) and their DNA barcodes are publicly available in the Barcode of Life Data System (BOLD), NCBI GenBank online databases and International Nucleotide Sequence Database Collaboration (INSDC).

## Introduction

Traditional approaches to biodiversity assessment have been revolutionised by DNA-based species identification (Hebert and Gregory 2005). DNA barcoding, which uses short standardised regions of DNA, called barcodes, to identify organisms (Hebert et al. 2003), has streamlined the species identification process, making it faster, more automated and less reliant on taxonomic expertise than classical methods (Hebert and Gregory 2005). DNA metabarcoding extends this to complex samples, allowing large-scale taxonomic identification and playing a crucial role in ecological studies, with great applicability to biodiversity conservation (Taberlet et al. 2012, Cristescu 2014, Taberlet et al. 2018). The development of this technique has been facilitated by advances in PCR technologies and high-throughput sequencing (HTS), which allows multiple DNA barcodes to be sequenced from a single sample (Taberlet et al. 2012, Forsman et al. 2022).

Metabarcoding is a particularly valuable tool for understanding trophic interactions (da Silva et al. 2019, de Sousa et al. 2019, Ando et al. 2020), as it enables dietary assessment at a higher taxonomic resolution and is faster than traditional methods and is applicable to non-invasive samples (Thuo et al. 2019, Gil et al. 2020). However, effective identification of taxa from environmental or dietary samples requires a reliable reference library of DNA barcodes (Pompanon et al. 2012, Cuff et al. 2022,Kress et al. 2015, Ferreira et al. 2019). In addition, libraries containing multiple barcode regions can further improve identification by overcoming PCR biases associated with marker selection (da Silva et al. 2019), as overlap between markers increases the likelihood of classifying all taxa in the sample (Fahner et al. 2016). For dietary studies, these accurate reference libraries are crucial for understanding the feeding habits and ecological roles of species (De Barba et al. 2014, Tercel et al. 2021).

**Figure 1.**
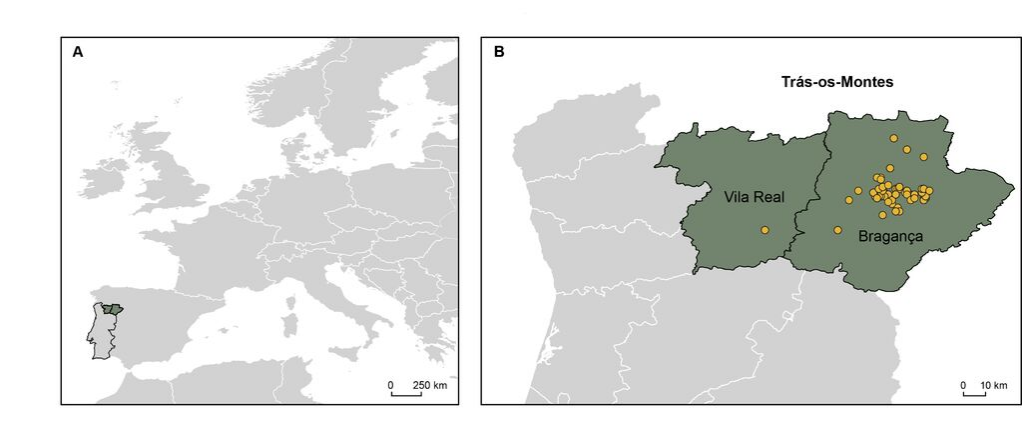
Geographical location of the study area. **A)** Location of Portugal within Europe; **B)** The Trás-os-Montes Region, showing the sampling points (in yellow) within the Vila Real and Bragança Districts.

The region of Trás-os-Montes is located in the mountainous northeast of Portugal, comprising the Bragança and Vila Real Districts (Fig. 1). This region is characterised by a diverse flora, with numerous endemic species, due to its diversity of habitats and the geochemistry of the substrate (Porto 2020). Two formations in eastern Bragança, for instance, are notable for their basic and ultrabasic igneous rocks, while a carbonate outcrop further east is also of interest (Aguiar and Alves 2020, Aguiar and Monteiro-Henriques 2020). For this reason, several studies have been carried out over the years on the local climate, geology and, especially, on plant diversity and ecology (Sequeira and Silva 1992, Aguiar et al. 2013, Preto et al. 2021). According to *Flora-On: Flora de Portugal Interactiva* (Flora-On: Flora de Portugal Interactiva 2024), of the 3082 plant taxa known for Portugal, 1706 (55.4%) occur in the Trás-os-Montes Region, of which 193 are endemic to the Iberian Peninsula and 22 are endemic to Portugal. Furthermore, Trás-os-Montes harbours 113 threatened species, 15 of which are critically endangered. In order to protect the unique diversity of this region, conservation efforts have been implemented through the establishment of natural parks, including Parque Natural de Montesinho and Parque Natural do Alvão (Queirós 2001). Despite the number of studies focused on this region, the flora of Trás-os-Montes continues to be enriched with new discoveries, of species previously unknown to occur in Portugal (e.g. Aguiar and Almeida 2006, Araújo 2021, Porto et al. 2024).

The present vascular plant dataset contributes to improving the molecular identification of the flora present not only in Portugal and the Iberian Peninsula, but also in other regions of the world where some species are also present. This DNA barcode reference library was generated in the framework of the InBIO Barcoding Initiative (IBI) and Agrivole project. The IBI project uses HTS technologies to build reference datasets of DNA barcodes from morphologically identified Portuguese specimens (Ferreira et al. 2018). The project has focused mainly on insects due to their importance in food webs and ecosystem functioning, with a total of 26 datasets already publicly avaiable (e.g. Ferreira et al. 2024, Pina et al. 2024). However, the project is now expanding to other groups, including plants. The Agrivole project aims to understand the responses of vole communities in the Trás-os-Montes Region to agroecosystem structure and agricultural practices and how this may affect the potential for pest outbreaks in orchards (i.e. olive groves) or the resilience of vole species of conservation concern in these habitats. The integration of IBI and Agrivole projects was crucial, as the Agrivole project focused on the use of faecal DNA metabarcoding analysis to determine the trophic niche of small mammals from Trás-os-Montes and, in order to obtain accurate and precise dietary estimates, a reliable plant DNA barcoding library for the region was required. As many species are still not represented in open sequence databases, a survey of local plants was essential to fill this gap. Therefore, this study created an accurate DNA reference database for the plants of the Trás-os-Montes Region of Portugal. To achieve this, all plant samples collected were morphologically identified by taxonomic experts and two molecular markers were amplified and sequenced: the chloroplast trnL (UAA) intron region using two primer sets (ef and gh; Taberlet et al. 1991, Taberlet et al. 2007) and the nuclear internal transcribed spacer 2 region, ITS2 using one primer set (UniPlant; Cheng et al. 2016). ITS2 is one of the most widely used molecular markers for plant barcode identification (Hollingsworth et al. 2011) and trnL is widely used in metabarcoding studies as it can be amplified even when using highly-degraded DNA (Taberlet et al. 2007), making these two regions complementary. This work contributes to the open dissemination and sharing of the distribution records and DNA barcodes of the vascular plant specimens that are part of our reference collection, thereby increasing the available public information. This database will not only serve the Agrivole project, but will also contribute significantly to other DNA metabarcoding studies and traditional molecular research, facilitating ecological, phylogenetic and conservation applications in the region and beyond.

## General description

### Purpose

This dataset was generated as part of the Agrivole project that aimed to investigate the ecological role of small mammals, particularly voles, in local agroecosystems of the Trás-os-Montes Region in north-eastern Portugal, in collaboration with the InBIO Barcoding Initiative (IBI), which uses HTS technologies to build reference datasets of DNA barcodes from morphologically identified Portuguese specimens. To achieve this goal, the Agrivole project investigated the trophic niche of these small mammals through DNA metabarcoding analysis of their faeces using high throughput sequencing methods. This required a reliable plant DNA barcoding library for the region to accurately and precisely infer the dietary taxa consumed by each species, for which collaboration with IBI was crucial. Therefore, this database aims to provide a reference database of DNA barcodes of the vascular plants of the Trás-os-Montes Region. This library will facilitate the DNA-based identification of plant species in the Agrivole project and other DNA metabarcoding and traditional molecular studies, such as environmental DNA (eDNA) monitoring, which has shown a growing trend in recent years (Cristescu 2014, Fahner et al. 2016, Forsman et al. 2022).

### Additional information

A total of 659 vascular plant samples were collected, of which 647 were morphologically identified to the most specific taxon (species, subspecies or variety), comprising 448 species belonging to 69 families (Suppl. materials 1, 2).

Of these, 632 samples were successfully barcoded, covering 435 species (Suppl. material 3). This represents 14% of the known plants in the Portuguese flora and 38.7% of the total known plant species in the study area (considering all the 10 x 10 km UTM squares where the samples are located; Flora-On: Flora de Portugal Interactiva (2024)). The relatively low representativeness is likely due to the high diversity of habitats that exist in the study area and that were not sampled, given the project's focus on the olive groves agroecosytems. To overcome this limitation, future sampling efforts should prioritise the remaining unexplored habitats, such as the basic and ultrabasic igneous rocks or the carbonate outcrops (Aguiar and Alves 2020, Aguiar and Monteiro-Henriques 2020).

With ITS2, 594 samples were successfully barcoded, 590 for trnL primer ef (trnL-ef) and 597 for trnL primer gh (trnL-gh), with 541 samples amplified for the three primer sets. This resulted in a total of 1781 barcode sequences for the sum of the three markers used (Suppl. materials 1, 4, 5, 6). All 69 families were represented; however, each primerset failed to amplify specific families most likely due to differences in primer specificity, with ITS2 lacking seven families (Aspleniaceae, Dennstaedtiaceae, Dioscoreaceae, Equisetaceae, Ophioglossaceae, Pteridaceae and Cytinaceae), trnL-ef one (Cytinaceae) and trnL-gh three (Montiaceae, Ophioglossaceae and Pteridaceae).

Of the 1781 barcode sequences, 1099 (61.7%) contain new information at different levels: 254 (13.6%, ITS2: 41, trnL-ef: 126, trnL-gh: 87) are completely new to GenBank and/or BOLD databases at the time of publication, 438 (24.6%, ITS2: 59, trnL-ef: 173, trnL-gh: 206) are new records for a given species and 407 (22.9%, ITS2: 187, trnL-ef: 206, trnL-gh: 14) provide additional information (e.g. different bp length, intraspecific genetic variability); the remaining 682 sequences (38.3%) are equal (100% identity) to sequences already publicly available for the identified species. Overall, this dataset represents a significant contribution to the genetic knowledge of vascular plants represented in public libraries and, specifically, within the scope of the Agrivole project, will be essential for studying the feeding ecology of small mammals and understanding their ecological interactions, including competition between species for food resources. This will provide insights into the responses of small mammal communities in the Trás-os-Montes Region to agro-ecosystem structure and agricultural practices and how this may affect the potential for pest outbreaks in orchards (i.e. olive groves) or the resilience of species of conservation concern in these habitats. Furthermore, this database is essential for future long-term monitoring studies of the plant communities in the study area and will assist the government authorities in the sustainable management of the National Parks (Queirós 2001).

## Project description

### Title

The InBIO Barcoding Initiative Database: contribution to the knowledge of DNA barcodes of the vascular plants of north-eastern Portugal.

### Personnel

Joana Paupério (project coordinator), João Queirós (junior researcher of project), Miguel Porto (plant specialist), Paulo Célio Alves (senior researcher in wildlife conservation and management), Pedro Beja (senior researcher in wildlife ecology), Ricardo Pita (project co-coordinator), Hélia Vale-Gonçalves (project technician), Catarina J. Pinho (project technician), Rodrigo Silva (Bachelor student in biology).

### Study area description

The region of Trás-os-Montes is located in the mountainous northeast of Portugal, comprising the Districts of Vila Real and Bragança (Fig. 1).

### Design description

Vascular plants were collected from traditionally managed olive grove agroecosystems and surrounding areas in north-eastern Portugal. Each distinct vascular plant species within each patch was collected, photographed and then mounted on herbarium sheets *in situ*. These were then morphologically identified by experts and a portion of the leaf tissue was used to obtain DNA barcodes.

## Sampling methods

### Study extent

Trás-os-Montes Region, Portugal

### Sampling description

The sampling protocol was designed to cover plants in areas where small mammals, particularly voles, were expected to occur, as defined under the Agrivole project (Barão et al. 2022). Plant samples were collected in the northeast of Portugal in August 2018, May–June 2019 and June 2020, from traditionally managed olive grove agroecosystems and their surrounding areas. Olive farming plays a critical role in local economy and has steadily expanded across the region (Duarte et al. 2006), currently covering approximately 15% of the land (unpublished data, Agrivole project). Young olive groves were used as a central point from which a 50 m buffer zone was drawn (Barão et al. 2022). Each distinct vascular plant species within each patch was collected, photographed and then mounted on herbarium sheets *in situ* with an assigned specimen number. A portion of leaf tissue was collected from each sample, placed in a paper envelope and then stored in a zip-lock bag with silica gel until DNA extraction. To minimise contamination between samples, tweezers and scissors were systematically sterilised with 100% ethanol under a flame. After morphological identification, all the specimens (vouchers) were deposited in the Herbarium of the Natural History and Science Museum of the University of Porto (MHNC-UP) with the codes PO-V72113–PO-V72772 (Suppl. material 1)

### Quality control

Collected samples were classified and revised by experts in vascular plant identification. The obtained DNA barcode sequences were compared against the GenBank and BOLD databases and the top hits were examined to identify potential PCR / sequencing errors, contaminations or misidentifications.

### Step description

1. Plant sampling

Vascular plants were collected in the northeast of Portugal in August 2018, May–June 2019 and June 2020, from traditionally managed olive grove agroecosystems and their surrounding areas. A leaf tissue sample was collected for DNA extraction.

2. Taxonomic identification

The morphological identification of the specimens was carried out, after herborisation, under a stereomicroscope, using mainly the dichotomous keys of *Flora Iberica* (Castroviejo 2020). The website *Flora-On: Flora de Portugal Interactiva* (Flora-On: Flora de Portugal Interactiva 2024) was used as a reference for the list of Portuguese vascular flora, as well as for visual validation and distribution of the taxa. Regarding nomenclature, the Angiosperm Phylogeny Website (Stevens 2024) was followed for suprageneric taxa, while scientific names were based on the Plants of the World Online (POWO 2024), except where specified otherwise. For some specimens, it was necessary to consult other works namely in cases of possible hybrids (Perez-Carro et al. 1985, Nesom 2006, Portela-Pereira et al. 2013, Krasnopoļska et al. 2020, Király et al. 2021, Martín-Gómez et al. 2022) or, in cases where the *Flora Iberica* has a different treatment of the taxon of interest than POWO (Pereira Coutinho 1913, Walters 1958, Guittonneau 1972, Alavi 1976, Tutin et al. 1980, Benedí 1986, Snogerup and Snogerup 2001, Uotila 2001, Desfayes 2004, Pavlickf 2007, Horjales et al. 2008, Molina et al. 2008, Blanca 2009, Arabaci et al. 2010, Cano-Maqueda and Talavera 2011, Rubido-Bará et al. 2011, Haerinasab and Rahiminejad 2012, Tison and De Foucault 2014, Särkinen et al. 2018, Karaismailoğlu 2019). Whenever possible, the specimens were identified at the infraspecific level.

3. DNA extraction

DNA was extracted for each sample using 20 mg of dry leaf material weighed into a 2 ml tube. To disrupt the thick plant cell walls, zirconia beads were added to the tube and placed on a mill for a minimum of 10 minutes. Lysis was performed by adding lysis buffer (2% SDS, 2% PVP 40, 250 mM NaCl, 200 mM Tris HCl, 5 mM EDTA, pH 8) and 10 µl of proteinase K (1 mg/ml) to the samples, followed by incubation at 63°C for 30 minutes. RNA was removed by adding 10 µl of RNAase A (10 mg/ml) followed by incubation at 37°C for 20 minutes. DNA was precipitated by adding 150 µl of potassium formate (KCOOH) (3 M), mixed by inversion of tubes, followed by incubation on ice for 25 minutes. A stepwise centrifugation was performed at increasing speed: 1 minute at 1000 rpm, at 2000 rpm, at 4000 rpm, at 8000 rpm and 10 minutes at 11000 rpm, followed by DNA binding using 550 µl of clear supernatant and 825 µl binding buffer (2 M guanidine hydrochloride in 95% ethanol). A maximum of 700 µl of binding mixture was added to a column, followed by centrifugation at 11000 rpm for 1 minute. This procedure was repeated for the remainder of the binding mixture. The column membranes were then washed twice with 80% ethanol (1 minute at 11000 rpm). Finally, two DNA elutions were performed by adding 50 µl of binding buffer (65^°^C 10 mM Tris-152 HCl, pH 8.3) to the column and centrifuging at 12000 rpm for 1 minute.

4. PCR amplification and library preparation

Polymerase chain reaction (PCR) amplification was performed focusing on two regions, the internal transcribed spacer of nuclear ribosomal DNA (ITS2), using the primer pair UniPlantF/UniPlantR (187–387 bp; Moorhouse-Gann et al. 2018) and the P6 loop of the plastid *trn*L (UAA) gene, which was amplified using two different sets of primers: ef (147–540 bp; Taberlet et al. (1991)) and gh (10–143 bp; Taberlet et al. (2007)). All primers contained Illumina overhang adaptors at the 5’ end.

PCR reactions were performed by mixing 5 µl QIAGEN Multiplex PCR Master Mix, 3.4 µl ultrapure water, 0.3 µl of each primer and 1 µl DNA extract. Cycling conditions consisted of an initial denaturation at 95ºC for 15 min, followed by 40 cycles of denaturation at 95ºC for 30 s, annealing at 54ºC for 30 s for ITS2 and 55ºC for 1 min for both *trn*L primers and extension at 72ºC for 30 s, with a final extension cycle at 72ºC for 10 min. A touchdown method was also used for some samples that initially failed amplification for ITS2 to increase the specificity of the PCR product. This was done using the cycling conditions previously presented, changing the annealing for 5 cycles with an initial temperature of 56ºC, reduced by 0.5°C for each cycle. Amplification success was visually verified by electrophoreses in a 2% stained gel agarose using 2 µl of PCR product.

A second PCR was performed to incorporate the Illumina sequencing adapters and P5 and P7 indices using 5 µl KAPA HiFi HotStart ReadyMix, 1 µl of index mix, 2 µl of ultrapure water and 2 µl of the previous PCR products diluted 1:10. Cycling condition consisted of an initial denaturation at 95°C for 3 minutes, followed by 10 cycles at 95°C for 30 seconds, at 55°C for 30 seconds and 72°C for 30 seconds, with a final extension at 72°C for 5 minutes. Indexing success was assessed by comparing the initial PCR product with the indexed product by electrophoresis. These were cleaned using 1.2 × AMPure^®^ XP beads, quantified using Epoch, diluted to 15 nM and pooled by marker. The three libraries were then quantified by qPCR and pooled to obtain a final library at 4 nM. The final library was sequenced on an Illumina MiSeq System, using a MiSeq V2 500-cycle reagent kit and considering a coverage of approximately 5,000 paired-end reads per sample and marker.

5. Bioinformatic analysis

The obtained sequences were bioinformatically processed using the OBITools software (Boyer et al. 2016). Paired-end reads were first aligned using the *illuminapairedend* command and discarded if the overlap quality was less than 40. Unaligned sequences were also removed using the *obigprep* command. Sequences were then dereplicated to unique sequences per sample using *obiuniq* and primer sequences were removed using *ngsfilter*. Sequence lengths were trimmed to the expected lengths mentioned above and sequences with less than two reads were removed. Clustering analysis was performed using *sumacluster* with 99% similarity between sequences to remove PCR and sequencing errors.

6. Data Analysis

To estimate the representativeness of the species collected in this study compared to the expected total diversity known for the Trás-os-Montes Region, we used as the reference dataset the list of plants described by family on the Flora-On website (Flora-On: Flora de Portugal Interactiva 2024).

To assess the contribution of this study as a source of new genetic resources (DNA barcodes), the DNA sequences generated were compared to the NCBI Nucleotide Database using the BLAST+ software (Camacho et al. 2009). The obtained sequences were used to create three local reference databases for each marker (ITS2, trnL-ef, trnL-gh) using the *makeblastDB* function. These databases were then compared to the NCBI online records using the *blastn* algorithm, with a word size of 28 for ITS2 and trnL-ef and a word size of 12 for trnL-gh due to its smaller size, applying minimum cut-offs of 90% identity and 80% coverage. Additionally, the ITS2 sequences were compared against BOLD databases using BOLD Identification Engine (Ratnasingham and Hebert 2007).

Sequences that did not match the morphological identification, at least at the family level, were considered errors and discarded. Sequences that matched with 100% coverage and identity to a different species within the same genus or family were considered new records for that species. Sequences were classified as completely new records for online databases if they showed identity percentages and query coverage below 90%. Sequences that correctly matched the morphological identification at the species level, but had a coverage of 80%–100% and/or an identity percentage between 98%–100%, were classified as new information records for that species.

## Geographic coverage

### Description

The study was carried out in the districts of Bragança and Vila Real, located in northeast of Portugal. In particular, vascular plants were collected from agroecosystems.

### Coordinates

-7.494 and -6.614 Latitude; 41.832 and 41.336 Longitude.

## Taxonomic coverage

### Description

**Figure 2.**
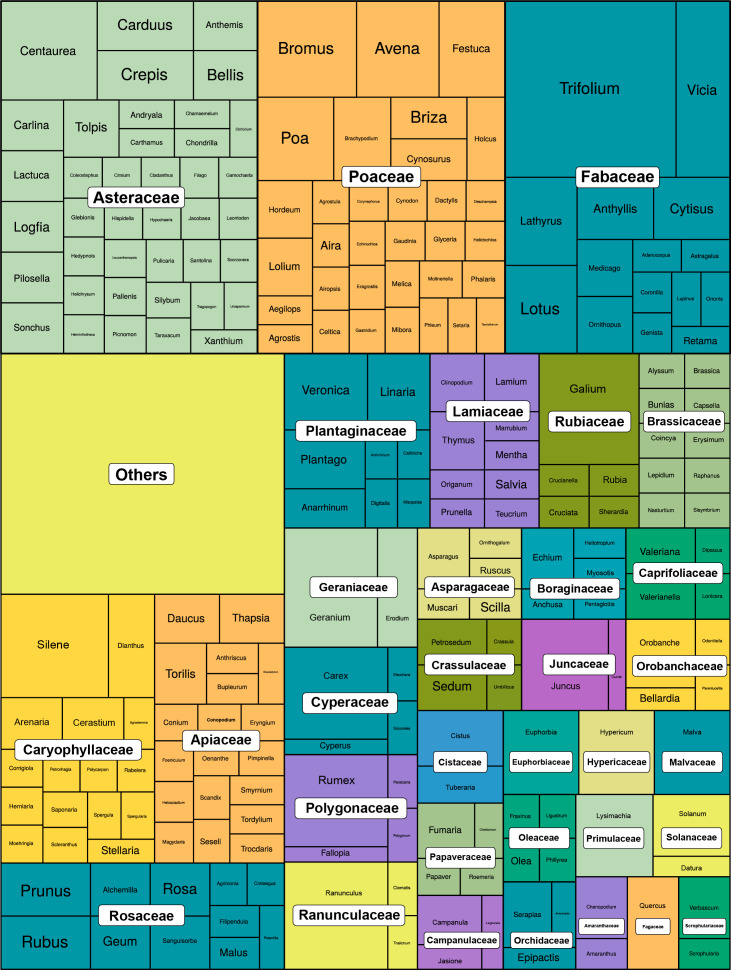
Treemap depicting the genus and corresponding plant families barcoded in this study for the Trás-os-Montes Region. The size of the squares is proportional to the number of species in each genus that were barcoded.

A total of 659 specimens were collected, of which 647 were morphologically identified to the most specific taxon (species, subspecies or variety). This dataset includes 448 taxa belonging to 69 families (Suppl. materials 1, 2). Of these, 632 samples were successfully barcoded, covering 435 species from 69 families (Fig. 2; Suppl. material 3). The families Poaceae (15%), Asteraceae (12%) and Fabaceae (12%) represented the highest percentages of samples collected and sequenced, while the remaining 66 families represented less than 5% each.

### Taxa included

**Table:** 

Rank	Scientific Name	Common Name
kingdom	Plantae	Plants
phylum	Tracheophyta	
class	Liliopsida	
order	Asparagales	
family	Amaryllidaceae	
family	Asparagaceae	
family	Orchidaceae	
order	Dioscoreales	
family	Dioscoreaceae	
order	Poales	
family	Cyperaceae	
family	Juncaceae	
family	Poaceae	
class	Lycopodiopsida	
order	Isoetales	
family	Isoetaceae	
class	Magnoliopsida	
order	Apiales	
family	Apiaceae	
order	Asterales	
family	Asteraceae	
family	Campanulaceae	
order	Boraginales	
family	Boraginaceae	
order	Brassicales	
family	Brassicaceae	
family	Resedaceae	
order	Caryophyllales	
family	Amaranthaceae	
family	Caryophyllaceae	
family	Montiaceae	
family	Plumbaginaceae	
family	Polygonaceae	
family	Portulacaceae	
order	Cucurbitales	
family	Cucurbitaceae	
order	Dipsacales	
family	Caprifoliaceae	
order	Ericales	
family	Ericaceae	
family	Primulaceae	
order	Fabales	
family	Fabaceae	
family	Polygalaceae	
order	Fagales	
family	Fagaceae	
family	Juglandaceae	
order	Gentianales	
family	Gentianaceae	
family	Rubiaceae	
order	Geraniales	
family	Geraniaceae	
order	Lamiales	
family	Lamiaceae	
family	Oleaceae	
family	Orobanchaceae	
family	Plantaginaceae	
family	Scrophulariaceae	
family	Verbenaceae	
order	Malpighiales	
family	Euphorbiaceae	
family	Hypericaceae	
family	Linaceae	
family	Salicaceae	
family	Violaceae	
order	Malvales	
family	Cistaceae	
family	Cytinaceae	
family	Malvaceae	
family	Thymelaeaceae	
order	Myrtales	
family	Lythraceae	
family	Onagraceae	
order	Piperales	
family	Aristolochiaceae	
order	Ranunculales	
family	Papaveraceae	
family	Ranunculaceae	
order	Rosales	
family	Cannabaceae	
family	Rosaceae	
family	Urticaceae	
order	Santalales	
family	Santalaceae	
order	Sapindales	
family	Anacardiaceae	
family	Rutaceae	
order	Saxifragales	
family	Crassulaceae	
family	Paeoniaceae	
family	Saxifragaceae	
order	Solanales	
family	Convolvulaceae	
family	Solanaceae	
order	Zygophyllales	
family	Zygophyllaceae	
class	Pinopsida	
family	Cupressaceae	
class	Polypodiopsida	
order	Polypodiales	
family	Aspleniaceae	
family	Dennstaedtiaceae	
class	Psilotopsida	
order	Ophioglossales	
family	Ophioglossaceae	
order	Equisetales	
family	Equisetaceae	
family	Pteridaceae	

## Temporal coverage

### Notes

Plant samples were collected in August 2018, May–June 2019 and June 2020.

## Collection data

### Collection name

Plantas vasculares - Herbário Português

### Collection identifier

Herbário da Universidade Do Porto (PO)

### Specimen preservation method

Dry

### Curatorial unit

PO-V72113 – PO-V72772

## Usage licence

### Usage licence

Creative Commons Public Domain Waiver (CC-Zero)

## Data resources

### Data package title

The InBIO Barcoding Initiative Database: contribution to the knowledge of DNA barcodes of the vascular plants of north-eastern Portugal.

### Number of data sets

1

### Data set 1.

#### Data set name

DS-IBPLT

#### Description

The data underpinning the analysis reported in this paper are deposited at BOLD, the Barcode of Life Data System, with the dataset name DS-IBPLT and GBIF, the Global Biodiversity Information Facility, at: https://doi.org/10.15468/exuufa.

**Data set 1.** 

Column label	Column description
materialSampleID	Unique identifier for the sample.
recordNumber	Identifier for the sample being sequenced in the IBI catalogue number at Cibio-InBIO, Porto University. Same as fieldNumber.
catalogNumber	Identifier for the specimen deposited in the Museum of the University of Porto (MHNC-UP).
institutionCode	The full name of the institution that has physical possession of the DNA samples.
occurrenceID	Global unique identifier for that sample.
kingdom	Kingdom name.
phylum	Phylum name.
class	Class name.
order	Order name.
subfamily	Subfamily name.
family	Family name.
genus	Genus name.
subpecies	Species nameSubpecies name.
scientificNameAuthorship	The authorship information for the sample.
identificationRemarks	Comments or notes about the taxonomic identification.
identifiedBy	Full name of the individuals who assigned the specimen to a taxonomic group.
typeStatus	Status of the specimen in an accessioning process.
recordedBy	The full names of the individuals or team responsible for collecting the sample in the field.
eventDate	Date of the sample collection.
countryCode	The full, unabbreviated name of the country where the specimens was collectedCode of the country where the specimens was collected.
lat	The geographical latitude (in decimal degrees) of the geographic centre of a location.
lon	The geographical longitude (in decimal degrees) of the geographic centre of a location.
county	The full, unabbreviated name of the county where the organism was collected.
municipality	The full, unabbreviated name of the municipality ("Concelho" in Portugal) where the specimen was collected.
verbatimLocality	The original textual description of the sampled location.
dynamicProperties	DNA barcoded sequences.
measurementDeterminedBy	The full name of the institution where DNA samples are stored.
datasetName	BOLD dataset code.

## Supplementary Material

31ABD5DC-F616-5109-88DD-3BB2F884C7F9 10.3897/BDJ.13.e142020.suppl1 Supplementary material 1 IBPLT DNA barcoding of Portuguese plants – Specimen details Data type Record information - specimen data Brief description The file includes information about all records in BOLD for the IBPLT DNA barcoding of Portuguese plants database. It contains collection and identification data. The data are as downloaded from BOLD, without further processing. File: oo_1218895.txt https://binary.pensoft.net/file/1218895 João Queirós, Rodrigo Silva, Catarina J. Pinho, Hélia Gonçalves, Ricardo Pita, Paulo C. Alves, Pedro Beja, Joana Paupério, Miguel Porto

B0596064-3EE7-541B-BE48-0B422600E9A8 10.3897/BDJ.13.e142020.suppl2 Supplementary material 2 IBPLT DNA barcoding of Portuguese plants – Specimen details – Darwin Core Standard Data type Record information - specimen data in Darwin Core Standard format Brief description The file includes information about all records in BOLD for the IBPLT DNA barcoding of Portuguese plants. It contains collection and identification data. The data are downloaded from GBIF (**https://doi.org/10.15468/exuufa**), without further processing. File: oo_1218896.txt https://binary.pensoft.net/file/1218896 João Queirós, Rodrigo Silva, Catarina J. Pinho, Hélia Gonçalves, Ricardo Pita, Paulo C. Alves, Pedro Beja, Joana Paupério, Miguel Porto

D33EAAC1-CECB-5514-83F9-356D829E6008 10.3897/BDJ.13.e142020.suppl3 Supplementary material 3 IBPLT DNA barcoding of Portuguese plants – List of specimens and accession codes Data type Record information, genetic accession codes Brief description List of species DNA barcoded in this project, including their original collection code, BOLD and GenBank accession codes for the three markers used (ITS2, trnl-ef and trnl-gh). In the trnl-gh column, (*) indicates sequences that did not meet the GenBank threshold of > 50 bp sequence length. These sequences are provided in Suppl. material 6. File: oo_1174828.csv https://binary.pensoft.net/file/1174828 João Queirós, Rodrigo Silva, Catarina J. Pinho, Hélia Gonçalves, Ricardo Pita, Paulo C. Alves, Pedro Beja, Joana Paupério, Miguel Porto

C4016CC9-9BCA-5855-9BD6-C05B2530BA83 10.3897/BDJ.13.e142020.suppl4 Supplementary material 4 IBPLT DNA barcoding of Portuguese plants – DNA sequences ITS2 Data type Genomic data, DNA sequences Brief description ITS2 sequences in fasta format. Each sequence is identified by the original collection code, BOLD ProcessID and GenBank/INSDC accession number, separated by a vertical bar. File: oo_1139790.fasta https://binary.pensoft.net/file/1139790 João Queirós, Rodrigo Silva, Catarina J. Pinho, Hélia Gonçalves, Ricardo Pita, Paulo C. Alves, Pedro Beja, Joana Paupério, Miguel Porto

1AA77C20-74EA-5D0A-8716-C85900D3908A 10.3897/BDJ.13.e142020.suppl5 Supplementary material 5 IBPLT DNA barcoding of Portuguese plants – DNA sequences trnL-ef Data type Genomic data, DNA sequences Brief description trnL-ef sequences in fasta format. Each sequence is identified by the original collection code, BOLD ProcessID and GenBank/INSDC accession number, separated by a vertical bar. File: oo_1139791.fasta https://binary.pensoft.net/file/1139791 João Queirós, Rodrigo Silva, Catarina J. Pinho, Hélia Gonçalves, Ricardo Pita, Paulo C. Alves, Pedro Beja, Joana Paupério, Miguel Porto

8983843C-EB31-56DF-88E3-940A2CB1029D 10.3897/BDJ.13.e142020.suppl6 Supplementary material 6 IBPLT DNA barcoding of Portuguese plants – DNA sequences trnL-gh Data type Genomic data, DNA sequences Brief description trnL-gh sequences in fasta format. Each sequence is identified by the original collection code, BOLD ProcessID and GenBank/INSDC accession number, separated by a vertical bar. (*) indicates sequences that did not meet the GenBank threshold of > 50 bp sequence length and therefore do not present accession numbers. File: oo_1139792.fasta https://binary.pensoft.net/file/1139792 João Queirós, Rodrigo Silva, Catarina J. Pinho, Hélia Gonçalves, Ricardo Pita, Paulo C. Alves, Pedro Beja, Joana Paupério, Miguel Porto
